# Optimizing time-in-target-range assessment for blood pressure: insights from a large-scale study with continual cuffless monitoring

**DOI:** 10.3389/fmed.2024.1396962

**Published:** 2024-06-26

**Authors:** Naomi D. L. Fisher, Tiago P. Almeida, David Perruchoud, Jay Shah, Josep Sola

**Affiliations:** ^1^Brigham and Women’s Hospital, Boston, MA, United States; ^2^Aktiia SA, Neuchâtel, Switzerland

**Keywords:** Blood pressure, time in target range, cardiovascular risk, cuffless blood pressure monitor, hypertension management

## Abstract

**Introduction:**

Blood pressure (BP) time-in-target-range (TTR) is an emerging predictor of cardiovascular risk. Conventional BP methods are fundamentally unable to provide an optimal assessment of TTR, using irregular measurements separated by lengthy intervals. We investigated the optimal duration and frequency for reliable, practical TTR assessment in clinical settings using continual monitoring.

**Methods:**

This retrospective study analyzed 2.3 million BP readings from 5,189 European home users (55 ± 11 years, 82% male, BMI 28.0 ± 5.8) using a cuffless BP monitor (Aktiia SA). Systolic BP (SBP) data over 15 consecutive days were assessed (29 ± 11 readings/subject/24-h; 434 + 132 readings/subject/15-day). Subjects were classified into risk-related TTR groups based on 15-day SBP data (24-h, target 90–125 mmHg; ≥6 daytime readings). Various measurement frequencies and durations (1–14 days; 24-h/daytime; 2, 4 or ≥ 6 readings/day) were compared to this reference. Two specific configurations paralleling ambulatory (“One-Day-24 h”) and home (“One-Week-Daytime”) BP monitoring were selected for detailed analysis.

**Results:**

The reference TTR classified 63.0% of the subjects as high risk, 19.0% intermediate, and 18.0% low. “One-Day-24 h” schedule inaccurately classified 26% of subjects compared to the reference TTR, and “One-Week-Daytime” schedule inaccurately classified 45%. Classification accuracy with both schedules was high for subjects with very low or very high reference TTR, but poor otherwise. Accuracy of ≥90% in TTR classification only occurred with 7 days of continual 24-h monitoring.

**Discussion:**

For the first time, with the benefit of a cuffless device that measures BP with sufficient frequency and duration, practical use of TTR is enabled as a potentially enhanced metric to manage hypertension.

## 1 Introduction

The rising prevalence of hypertension, poor control rates and tighter blood pressure (BP) goals have propelled intense innovations in hypertension management (1). One new approach involves calculating BP time in target range (TTR), shown to be a strong independent predictor of cardiovascular event risk (2–6). BP TTR is poised to become a cornerstone in hypertensive management and risk stratification, but to date this new metric has been derived only from infrequent measures. Clarity around frequency and duration of BP measurements needed to calculate TTR for use in clinical practice is lacking (7).

The few published studies providing an estimate of TTR relied on intermittent snapshots of BP data spaced over irregular and lengthy intervals. TTR has been measured by office, ambulatory or home BP monitoring (OBPM, ABPM and HBPM), all of which are inherently limited in their ability to produce data for optimal and precise assessment of TTR (2–6). These modalities all use cuff-based oscillometric sphygmomanometers, and all cardiovascular outcomes data are based on measurements with these cuff-based technologies. But they can be inconvenient, burdensome, and sometimes uncomfortable. Additionally, they provide only episodic readings, and are prone to faulty measurements by both providers in office and patients at home (8). Consequently, cuff-based BP modalities do not facilitate the use of TTR in clinical practice, nor enable the full potential of TTR for hypertensive risk stratification in clinical practice or in research.

BP TTR, as a concept, has emerged concurrently with the evolution of cuffless BP monitoring devices (9, 10). Some cuffless BP devices can overcome the inherent limitations of cuff-based BP monitors, enabling continual out-of-office BP monitoring (8). Recent studies have demonstrated the clinical relevance of remote monitoring and cuffless BP devices under different conditions, prompting this investigation of how best to measure BP TTR using a cuffless BP device (9–11).

In a cohort of adults wearing a cuffless BP monitor in the outpatient setting, we sought to compare different durations and frequency of BP measurement to determine how best to assess BP TTR reliably in clinical practice.

## 2 Methods

### 2.1 Study population

This retrospective study examined 2,252,224 BP readings from 5,189 subjects from the UK, Germany and Switzerland. All subjects voluntarily purchased and wore a validated CE-marked, over-the-counter cuffless wrist BP monitor that measures BP optically following initialization with an oscillometric BP device (Aktiia SA, Neuchâtel, Switzerland) (9–12). The Aktiia monitor is a novel device that received regulatory approval based on adapted standards for validation; note that new standards to harmonize the validation of cuffless devices are still in preparation (9–12). All subjects provided permission to use their anonymous and/or aggregated data for research purposes. Diagnoses of hypertension and medications taken were unknown.

### 2.2 Data collection

BP data were continually recorded by the Aktiia monitor between January 2021 and September 2022. Optical sensors embedded in a small bracelet passively, automatically and continually collect photoplethysmography (PPG) signals acquired on the user’s wrist (approximately hourly) (9). Whenever the smartphone application is accessed by the user, signals are transferred via Bluetooth from the bracelet to the smartphone application, and forwarded to Aktiia’s cloud server. Following the initialization process, pulse wave analysis is applied to the PPG signals to estimate BP, which is displayed back in the smartphone application. The PPG signals and respective BP values are stored on the Aktiia’s cloud server and can be accessed remotely for retrospective analyses.

The Aktiia device has been validated in an investigation involving 86 participants and 327 paired BP measurements using an extended ISO 81060-2 protocol (11). Additionally, the Aktiia device showed similar performance compared to double auscultation in an elderly cohort, with 469 paired measurements from 35 subjects, per the ISO 81060-2 protocol (13). A study comparing readings from the Aktiia device with arterial line BP measurements in 31 subjects demonstrated strong correlation (14), and several studies have shown the Aktiia device provides daytime BP measurements equivalent to those obtained by 24-h ABPM (15, 16).

In the present work, SBP data from one full month were downloaded from the Aktiia’s cloud server for each subject. The number of measurements depended on a person’s activity level; BP reading is only performed if the wrist is still for 30 s. To avoid including days with fewer measurements in more active people, the first consecutive 15-day period was selected for each subject during which there were at least six daily daytime readings. For outlier rejection, days with more than one hundred daily daytime readings were removed from the analysis (0.05% of all analyzed days). This approach resulted in an average of 29 measurements/subject per 24-h (Supplementary materials), and 434 readings/subject for the 15-day reference period (Table 1). A similar analysis was conducted considering the first consecutive 30-day period and, although the number of analyzable subjects was smaller (3,577 instead of 5,189), the results were very similar. Therefore, the present work presents analyses from the 15-day period.

**Table 1:** Demographic and clinical characteristics of the study population (*n* = 5,189).

Age, years	55.3 ± 11.0	
Male	4,232 (82%)	
BMI, kg/m^2^	28.0 ± 5.8	
First cuff-based measured SBP, mmHg	134.7 ± 15.0	
≤120	15.1%	
≥130	62.4%	
≥140	32.8%	
First cuff-based measured DBP, mmHg	83.6 ± 10.5	
≤70	9.5%	
≥90	27.1%	
≥100	6.0%	
Number of BP readings	**Total**	**Per subject**
Reference TTR (15 days)	2,252,224	434.1 ± 132.0
“One-Day-24 h” schedule	153,367	29.6 ± 12.2
“One-Week-Daytime” schedule	707,367	136.3 ± 49.8
SBP values, mmHg		
Reference TTR (15 days)	**133.8 ± 15.8**	**vs. (p-value)**
“One-Day-24 h” schedule	133.9 ± 15.8	0.828
“One-Week-Daytime” schedule	135.2 ± 15.8	<0.0001

Initial SBP and DBP values were measured with a fully validated oscillometric device on the first day of the investigated 15 days, and used for initialization (12).

### 2.3 Dataset for TTR estimation and a reference BP TTR

A reference TTR was calculated for each subject using every systolic BP (SBP) obtained over the selected 15-day period (day and night), and defined as the percentage of SBP readings within the target range, set between 90 and 125 mmHg in accordance with 2017 ACC/AHA guidelines for 24-h goal (17). The lower limit of 90 mm Hg was chosen based on the commonly accepted definition of hypotension (18, 19). Based on their respective TTR, subjects were classified into groups previously shown to correlate with cardiovascular risk: Group A, 0% ≤ TTR < 25%; Group B, 25% ≤ TTR < 50%; Group C, 50% ≤ TTR < 75%; Group D, TTR ≥ 75% (3, 4).

### 2.4 Test of different methods for TTR estimation to compare to the reference

Using the 15-day dataset, different combinations of frequencies and duration of BP measurements to calculate TTR were examined with multiple variables:

*Duration of measurement period*: TTR was calculated with eleven different lengths of monitoring, ranging from 1 to 14 consecutive days (1, 2, 3, 4, 5, 6, 7, 8, 10, 12 and 14 days).

*Daytime-only BP data*: TTR was calculated using only daytime SBP readings (6 am-10 pm), with target range 90–130 mmHg in accordance with the 2017 ACC/AHA guidelines for HBPM (17).

*24-h BP data*: TTR was calculated using SBP readings over the course of 24-h, with appropriately tighter target range 90–125 mmHg (17).

*Frequency of measurements per 24-h*: TTR was calculated with three different frequencies of daytime only data (2, 4, or ≥ 6 daytime readings/day); and with three different frequencies of 24-h data (2, 4, ≥6 readings/day). In total, 33 different configurations were created to calculate TTR using daytime-only SBP data, and 33 using 24-h data (11 different lengths of monitoring, each with three different frequencies of daily readings).

Two specific configurations were selected for detailed TTR analysis compared to the reference because of their parallels with common BP measurement schedules. “One-Week-Daytime” TTR was calculated from daytime readings during the first full week of the 15-day range, chosen for its similarity to HBPM. “One-Day-24 h” TTR was calculated from readings during the first 24-h period of the 15-day range. To pursue comparison with ABPM reporting, two subsets of the “One-Day-24 h” schedule were further analyzed, calculating TTR in people whose dataset satisfied minimum published criteria for a valid 24-h ABPM: at least 26 readings (*n* = 3,571), and at least 20 daytime and 7 night-time readings (*n* = 2,127) (8, 20).

In all cases, TTR was calculated as the percentage of SBP readings within the target ranges defined by either daytime (90–130 mmHg) or 24-h (90–125 mmHg) BP data.

### 2.5 Statistical analysis

TTR calculated with each of the tested configurations was classified into four BP risk groups (groups A, B, C and D) and compared to the classification of BP risk group performed by the reference TTR. Confusion matrices were created to demonstrate the distribution of BP risk group classification by the “One-Day-24 h” and “One-Week-Daytime” schedules compared to classification by the reference TTR, with performance assessed by sensitivity (hit rate). Reasons for favoring sensitivity over accuracy are provided in Supplementary materials, together with accuracy and F1-score.

ANOVA was conducted for a multiple comparison test of the mean of SBP values for the 15-day reference, “One-Day-24 h” and “One-Week-Daytime” periods. *p*-values less than 0.05 were considered statistically significant.

## 3 Results

There was an average of 29.01 ± 11.4 daily 24-h readings per subject, including 19.28 ± 8.8 daytime and 9.80 ± 4.4 night-time readings. One hundred percent of the days had six or more measurements and 91.2% of the nights had six or more measurements. Subjects were predominantly overweight and male (Table 1). Average 24-h BP was 133.8 ± 15.8 mmHg; 60% had hypertensive first SBP readings (≥130 mmHg). The reference TTR classified 63.0% of the subjects as group A, 10.3% as group B, 8.7% as group C and 18.0% as group D. Including all readings for each case, there was no difference between the average SBP during the 15-day period and the “One-Day-24 h” period (*p* = 0.828), while the “One-Week-Daytime” average SBP was significantly higher than both the 15-day period and “One-Day-24 h” (*p* < 0.0001 for both), Table 1. Further details regarding SBP yield and values for the reference TTR and selected configurations are reported in Supplementary materials.

### 3.1 Selected BP configurations for TTR investigation

**Figure 1:**
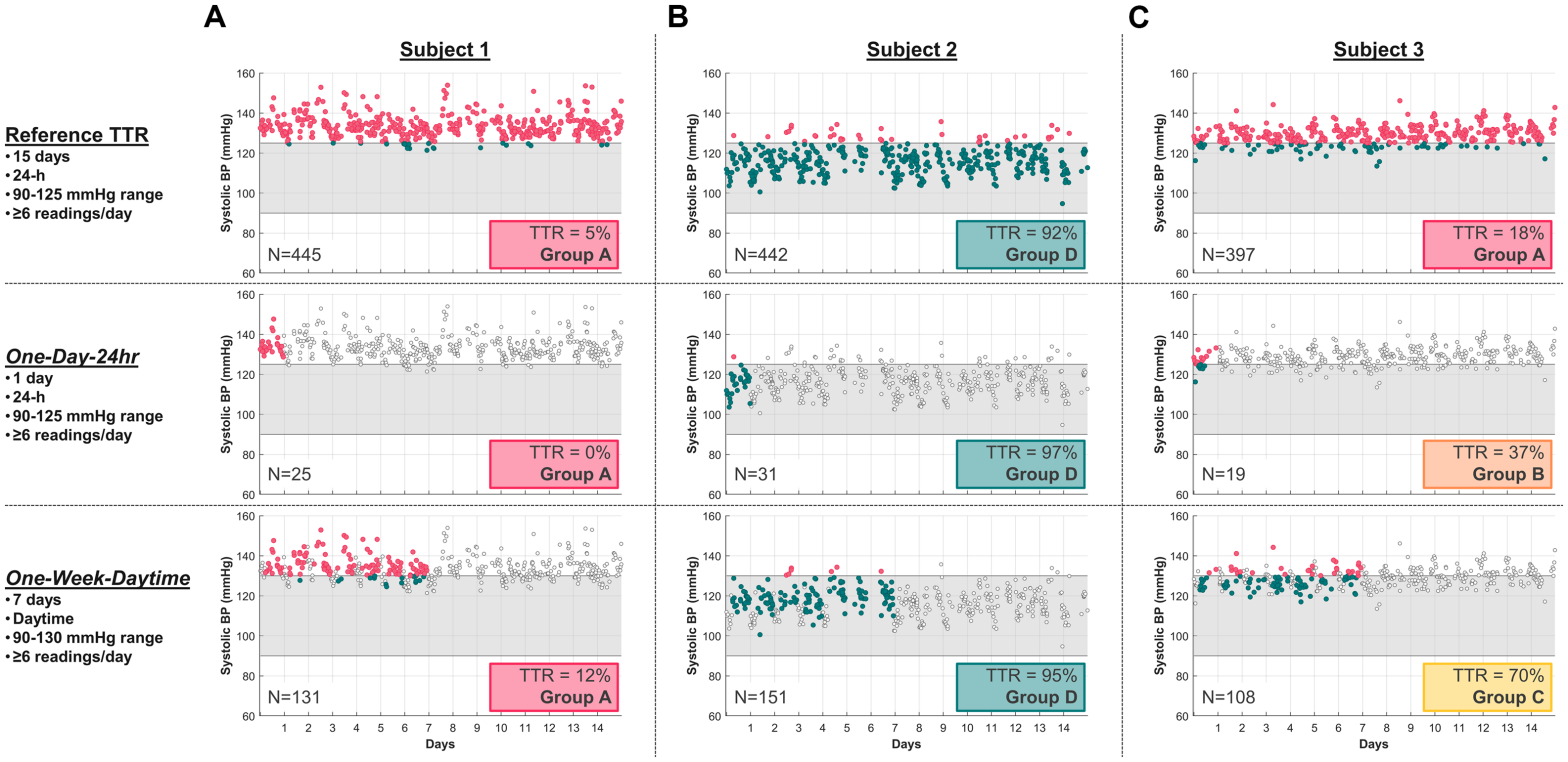
Typical SBP readings from three study subjects using the Aktiia monitor, comparing their 15-day reference TTR with a selected “One-Day-24 h” and a “One-Week-Daytime” schedule. SBP data points outside the respective target ranges (grey region) are marked in red, within range in green, and not included in the TTR analysis as light grey. **(A)** Subject 1 illustrates consistently low TTR for all three modalities (Group A), with 5% for the reference, 0% for “One-Day-24 h,” and 12% for “One-Week-Daytime” schedule. **(B)** Subject 2 illustrates consistently high TTR for all three modalities (Group D), with 92% for reference, 97% for “One-Day-24 h” schedule and 95% for “One-Week-Daytime” schedule. **(C)** Subject 3 illustrates conflicting TTR group risk classification across modalities. *N* = number of readings.

Among all subjects, TTR calculated by either “One-Day-24 h” or “One-Week-Daytime” schedules compared to the reference TTR was highly variable. Figure 1 shows typical SBP readings from three study subjects collected over 15 days using the Aktiia monitor, and their respective TTR according to the reference and to “One-Day-24 h” and “One-Week-Daytime” schedules. Figure 1A illustrates a subject with consistently low TTR, with the reference and the “One-Day-24 h” and “One-Week-Daytime” schedules (risk group A). Figure 1B illustrates a subject with consistently high TTR, using the reference and the “One-Day-24 h” and “One-Week-Daytime” schedules (risk group D). Figure 1C highlights the impact of different frequency and duration of BP monitoring in estimating TTR. The reference TTR classified the subject as high risk (group A), while the “One-Day-24 h” and “One-Week-Daytime” schedules classified the same subject as BP risk groups B and C, respectively.

**Figure 2:**
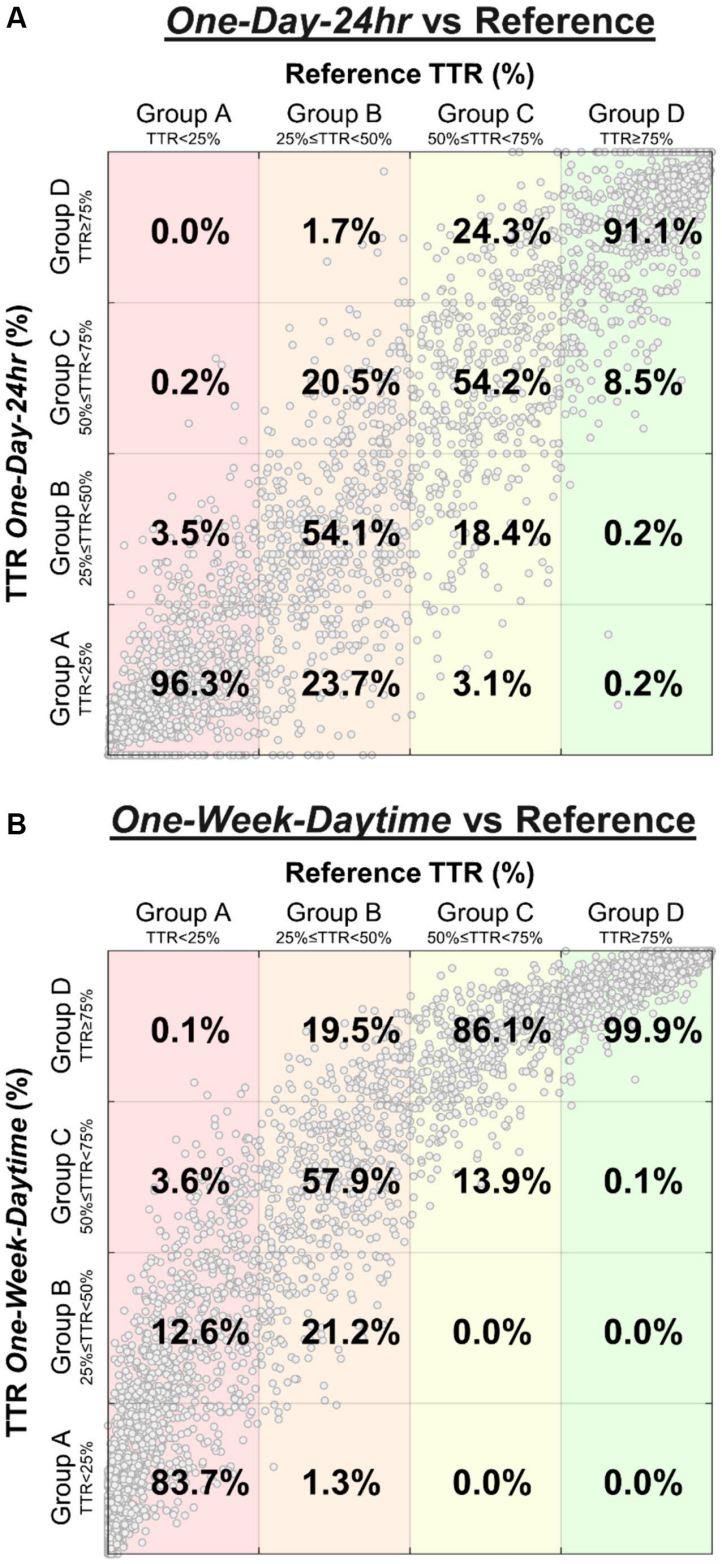
Confusion matrices highlight the distribution of BP risk group classification performed by “One-Day-24 h” schedule **(A)** and “One-Week-Daytime” schedule **(B)** compared to the reference TTR. The percentages represent the sensitivity for each class (percentages sum to 100% in each column). Large clusters found in groups A and D represent many subjects who were either consistently within or outside target range, and in concordance with the reference TTR. For example, of all subjects classified as group A by the reference (**A**, first column), 96.3% were correctly classified by the “One-Day-24 h” schedule, while 3.5% were misclassified as group B, and 0.2% as group C. Similarly, of all subjects classified as group A by the reference (**B**, first column), 83.7% were correctly classified by the “One-Week-Daytime” schedule, while 12.6% were misclassified as group B, 3.6% as group C, and 0.1% as group D.

Classification sensitivities comparing the “One-Day-24 h” and “One-Week-Daytime” schedules with the reference TTR differed by group, highlighted by confusion matrices (Figure 2). Sensitivities were highest in groups A and D; most subjects in these groups were either consistently within or outside target range, and in concordance with the reference TTR. The correspondence between reference TTR and “One-Day-24 h” schedule (Figure 2A) was stronger than with “One-Week-Daytime” schedule (Figure 2B).

### 3.2 Optimal BP monitoring strategy for TTR investigation

**Figure 3:**
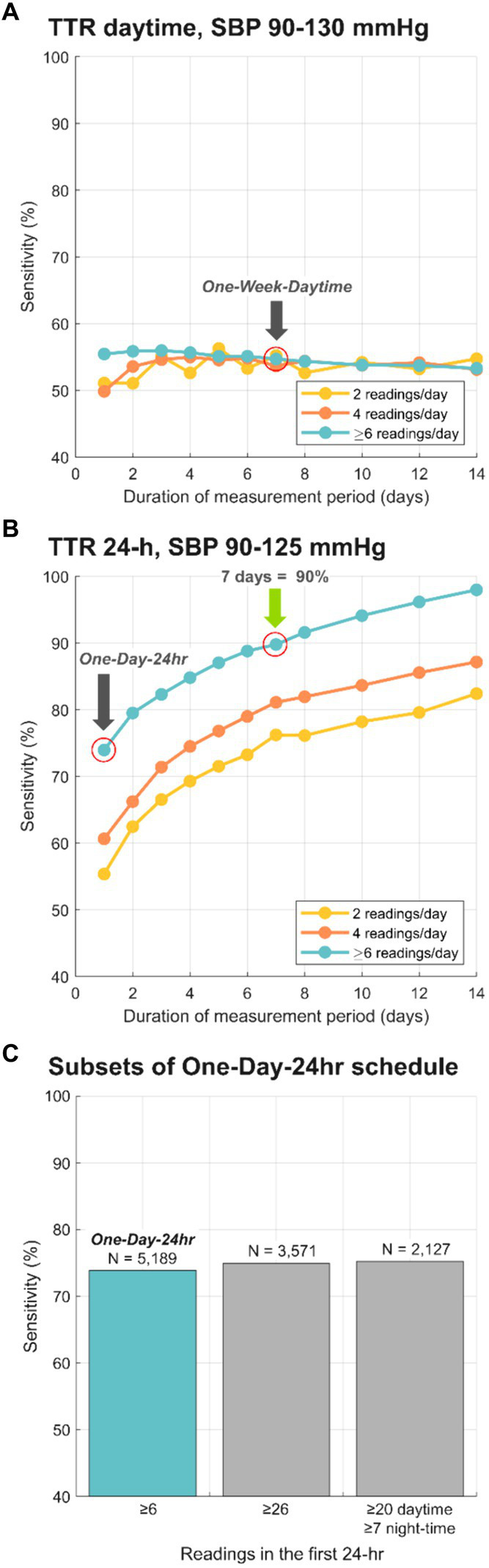
Sensitivity of BP risk group classification, comparing the reference TTR to different schedules for BP monitoring. **(A)** depicts sensitivity for TTR calculated with daytime only SBP for a range of days, and only 2, only 4 and at least 6 readings/day. **(B)** depicts sensitivity for TTR calculated with 24-h SBP data for a range of days, and only 2, only 4 and at least 6 readings/day. Grey arrows highlight the sensitivity for selected layouts: 73.9% for “One-Day-24 h” schedule and 54.7% for “One-Week-Daytime” schedule. Green arrow shows the minimal schedule (7 days; 24-h SBP data) that reached 90% sensitivity compared to the reference TTR. **(C)** Sensitivities for subsets of “One-Day-24 h” schedule vs. the 15-day reference. Sensitivity for One-Day TTR calculated with the original dataset (*N* = 5,189, ≥6 readings/day) was 73.9%. Sensitivity for One-Day TTR calculated with 3,571 subjects with at least 26 readings in the first 24-h was 74.93%, and for those 2,127 subjects with at least 20 daytime and 7 night-time readings was 75.25%.

Finally, we examined the effect of including different quantities of daily readings over differing ranges of days on the classification of TTR (Figure 3). Figure 3A illustrates the sensitivities of daytime TTR values calculated using daytime only SBP readings calculated over a range of days and number of readings per day. The sensitivity for “One-Week-Daytime” schedule (7 days; target range 90–130 mmHg; ≥6 readings/day), chosen for its parallels to HBPM, is highlighted (54.7%). Measurements taken only during the daytime resulted in suboptimal classifications regardless of duration of monitoring and number of readings per day. Conversely, classification performance of 24-h monitoring improved with both more readings and longer duration of monitoring (Figure 3B), with a sensitivity of 90% after one week of monitoring with at least 6 measurements/day. The sensitivity for the “One-Day-24 h” was 73.9% Additional classification performance metrics (accuracy, F1-score, kappa and positive predictive value) have been included in Supplementary materials. Only 2.9% of nights had fewer than 3 nocturnal readings per day, and the analysis performed excluding days with fewer than 3 nocturnal readings led to essentially identical results.

The “One-Day-24 h” schedule was chosen for its parallels ABPM. Additionally, TTR according to two subsets of the “One-Day-24 h” schedule were calculated: these included only patients whose dataset satisfied minimum published criteria for valid 24-h ABPM interpretation (8, 20). The first subset included 3,571 subjects with at least 26 readings in the first 24-h, which resulted in 74.93% sensitivity (vs. the 15-day reference). The second subset included 2,127 subjects with at least 20 daytime and 7 night-time readings in the first 24-h, which resulted in 75.25% sensitivity (vs. the 15-day reference). Figure 3C shows the sensitivity for “One-Day-24 h” schedule and subsets. To eliminate the unlikely possibility of BPs being higher on the first day of measurement because of patient alarm, the same “One-Day-24 h” schedule analyses were run using data from the second day rather than the first. There was no difference in sensitivity (75–76%) for both 26 mixed and at least 20 daytime, 7 nighttime values.

## 4 Discussion

TTR is emerging as a new metric of hypertension control, with the potential to be a better predictor of risk than static BP measurements. This study demonstrated that TTR is markedly impacted by measurement frequency and duration, and that at least one week of 24-h continual monitoring was needed to classify TTR with 90% sensitivity. The contributions of frequency of measurements and duration of measurement were unequal. Even very frequent monitoring over the course of one 24-h period was not sufficient to calculate TTR reliably. Collecting the requisite number and density of measurements is practically achievable only with continual cuffless BP monitors that can generate datasets allowing for TTR classification in real-world clinical use. This report is the first to analyze the ability of different continual BP measurement strategies to assess TTR.

### 4.1 A new method to assess BP “control” in clinical practice

TTR originated in the realm of anticoagulation, where percentage of international normalized ratio values in range was found to be an easy, practical metric that correlated well with outcomes and risk (21, 22). Later, “time in range” was adopted by diabetes specialists, when the development of continuous glucose monitors provided data to enable this novel measure to aid in the control of blood sugar. Time in range is widely accepted as appropriate and useful, both as a target and as an outcome (23).

TTR in the world of hypertension control is a more recent application. In 2017, Doumas et al. (3) were the first to propose SBP TTR as a novel measure of hypertension management, analyzing clinic BPs entered into the Veterans Administration’s (VA) systemwide database. Following patients for ten years, they reported an inverse and gradual association between TTR and all-cause mortality. Chung et al. (2) examined primary care records of people with newly identified hypertension in England, and showed that higher TTR was associated with lower risk of incident cardiovascular diseases over five years. In a *post hoc* analysis of SPRINT, Fatani et al. (4) reported a significant association of TTR with major adverse cardiovascular events: SBP TTR below 50% was associated with double the cumulative incidence of events. The authors showed TTR to be a robust independent predictor of cardiovascular events even after multivariate adjustments for SBP and BP variability. Examining results from SPRINT and from ACCORD, Buckley et al. (5) provided evidence associating higher SBP TTR with lower risk of adverse kidney and cardiovascular events in adults with hypertension. And in a secondary analysis of the TOPCAT trial, where TTR was calculated using linear interpolation in patients with heart failure with preserved ejection fraction, greater time in SBP target range was associated with decreased risk of cardiovascular outcomes and mortality events beyond BP (6). Despite their valuable contributions, these investigations were restricted by limitations imposed by cuff-based BP monitoring devices, and refinement of the assessment of TTR has been anticipated (24).

Additionally, methods for estimating TTR have relied on sparse measurements – usually one to three annually, and at most 4.5 per year in data drawn from the SPRINT study. Compared to this approach, the cuffless monitoring device used in our study offers dramatically more data points. Specifically, the cuffless device garnered an average of 434 individual BP readings per person within the initial 15 days, and 686 readings in the first month alone.

For decades, hypertension guideline targets relied on office BPs. The importance of out-of-office measurements is now widely recognized, and guidelines recommend management supported by ABPM and/or HBPM (8, 17). Specifically, two selected BP monitoring configurations intended to parallel the schedules of ABPM (“One-Day-24 h”) and HBPM (“One-Week-Daytime”) were analyzed, both with sufficient readings to meet published recommendations (8, 17). They each resulted in a significant portion of misclassified TTR risk groups. Subjects with intermediate risk (groups B and C) were more vulnerable to misclassification, while subjects with consistently high or low TTR were less so. Somewhat surprisingly, a significantly higher frequency of monitoring in the initial 24-h period (compared to ≥6 readings) did not substantially improve the sensitivity of TTR classification.

### 4.2 Clinical perspectives

To date, TTR has only been estimated via ABPM, HBPM (2–4), or OBPM (5, 6), using episodic snapshots of BP measurements; our data highlight the limitations in describing TTR accurately until now. Real-time TTR calculation by an easy-to-use device is a powerful yet simple tool that can deliver meaningful and actionable data to providers. Continual cuffless BP monitors, like the device used for this study, can readily measure TTR with markedly enhanced temporal resolution, resulting in better BP risk group classification, bringing TTR closer to clinical practice.

TTR calculated from SBP data sets that paralleled the frequency and duration of routine HBPM and ABPM were shown to be insufficient to classify TTR accurately and reliably. Our results indicate that, with one week of continual measurements, a novel wrist BP monitor can be used to calculate TTR. This type of monitor may better support remote BP management programs, which have been shown to optimize guideline-directed therapy, reduce cardiovascular risk, and minimize in-person visits among diverse populations (25). TTR can potentially be an effective and motivating component of hypertension self-management, providing feedback directly to patients. Patient engagement with TTR data could potentially improve control, by encouraging medication adherence and/or lifestyle intervention.

### 4.3 Limitations

The present work represents a retrospective analysis and was limited to 15 days of BP data in a predominantly male cohort. The hypertension status of subjects, whether they were taking medications (and which), and comorbidities were unknown. However, this information should not affect the ability of BP monitoring strategies to calculate TTR accurately and practically. The SBP target ranges used for the calculation of TTR by cuffless measurements are based on US guidelines delineated for cuff-based BP technologies. No SBP thresholds for TTR calculation are currently formalized and agreed upon using SBP data generated from cuffless BP devices. Future long-term studies employing cuffless BP devices will help address correlations between TTR and cardiovascular outcomes, as well as to define references and optimal targets for TTR.

The Aktiia device has been validated in multiple studies (11, 13–16). In contrast to excellent correlation of daytime BPs, there is a known difference in scaling of measurement of nocturnal readings with cuffless devices (9, 15, 16, 26, 27). However, this does not pertain to the primary focus of our work, to explore the optimal time schedule of BP monitoring for TTR estimation using continual monitoring. In this context, any discrepancies in scaling nocturnal BP readings would be uniformly present across all monitoring schedules, including the reference TTR, allowing for a consistent comparison. Consequently, while absolute values of BP readings might vary due to device-specific characteristics, relative changes and patterns vital for optimal monitoring period determination would remain unaffected. This ensures the study’s conclusions are based on a consistent comparison across various monitoring schedules.

The analysis in the present work focused on SBP, as previous studies have demonstrated that higher SBP is associated with coronary atheroma progression, coronary heart disease, stroke, cardiovascular mortality, and all-cause mortality (4). Additionally, SBP has been shown to be a better target for treatment, particularly among middle aged or older individuals (3).

Although guidelines for HBPM require duplicate morning and evening measurements, organizing the data into morning and evening periods was not feasible due to limitations to the study’s design. First, the Aktiia monitor does not support spot checks. Instead, it collects PPG data whenever the subject’s wrist is still for 30 s (approximately one measurement per hour). Second, this study does not represent a clinical trial with fixed schedules for data collection. Instead, it utilized data from real-world users, collected as they carried out their normal routines. The Aktiia monitor gathered data (roughly once per hour) whenever users remained still for 30 s. To maximize the statistical power of the data, three different frequencies of daytime-only readings were tested (2, 4, or ≥ 6 readings per day), without distinguishing between morning and evening periods.

## 5 Conclusion

In the present work, we have shown that TTR is highly dependent on the schedule and strategy used to measure SBP over time. At least one week of 24-h continual monitoring was needed to identify a subject’s BP risk with TTR at 90% sensitivity, which can only be achieved in practice with cuffless BP devices. Our results suggest that continual cuffless BP monitoring enables rapid and practical assessment of SBP TTR, an emerging metric of hypertension control.

Cuffless BP technologies represent a dramatic shift in the paradigm of BP monitoring and hypertension management. The employment of cuffless BP devices in future studies should allow improved assessments of hypertension control, and their use in clinical practice and research may innovate hypertension management.

## Data availability statement

The datasets presented in this article are not readily available because the generated datasets used in this work are proprietary to Aktiia SA. Requests to access the datasets should be directed to TA, publication@aktiia.com.

## Ethics statement

Ethical approval was not required for the study involving humans in accordance with the local legislation and institutional requirements. Written informed consent to participate in this study was not required from the participants or the participants’ legal guardians/next of kin in accordance with the national legislation and the institutional requirements.

## Author contributions

NF: Writing – review & editing, Writing – original draft, Methodology, Investigation, Conceptualization. TA: Writing – review & editing, Writing – original draft, Visualization, Software, Methodology, Investigation, Formal analysis. DP: Writing – review & editing, Visualization, Software, Methodology, Investigation, Formal analysis, Data curation, Conceptualization. JaS: Writing – review & editing, Methodology, Investigation, Conceptualization. JoS: Writing – review & editing, Visualization, Software, Methodology, Investigation, Formal analysis, Data curation, Conceptualization.
