# Supplementary material for: Optimizing time-in-target-range assessment for blood pressure: insights from a large-scale study with continual cuffless monitoring
Source: Front Med (Lausanne). 2024 Jun 26;11:1396962. doi: 10.3389/fmed.2024.1396962 (PMC11233795; doi:10.3389/fmed.2024.1396962)
Supplement: Supplementary file 1 [file Data_Sheet_1.DOCX]

Supplementary Material

Optimizing Time-in-Target-Range Assessment for Blood Pressure: Insights from a Large-Scale Study with Continual Cuffless Monitoring

Naomi DL Fisher^1^, Tiago P Almeida^2^, David Perruchoud^2^, Jay Shah^2^, Josep Sola^2^

^1^Brigham and Women’s Hospital, Boston, MA (N.D.L.F.)

^2^Aktiia SA, Neuchâtel, Switzerland

# Methods

## TTR for ‘One Day 24-hr' and ‘One Week Daytime’ schedules

Data from the first 15 consecutive days within the period between January 2021 and September 2022 were used to calculate the reference TTR, data from the first day to calculate TTR based on ‘One-Day-24hr schedule,’ and data from the first 7 days to calculate ‘One-Week-Daytime’ schedule, resulting in some overlap of BPs among the schedules investigated (detailed in Figure S1.)


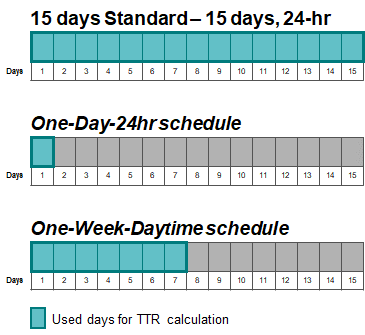


**Supplementary Figure 1.** Layout of data collection for TTR reference and the two selected configurations investigated in the present study.

## Sensitivity versus accuracy

### Accuracy

Accuracy is the ratio of all correctly predicted values *versus* the entire population (actual positive P and actual negative N), considering the correctly predicted presence (true positive, TP) or absence (true negative, TN) of a condition:

| $Accuracy=\frac{TP+TN}{P+N}$ | (1) |
| --- | --- |

### Sensitivity

Sensitivity (or hit rate) is the ratio of all correctly predicted presence of a condition (TP) versus the real positive cases in the data (actual positive):

| $Sensitivity=\frac{TP}{P}$ | (2) |
| --- | --- |

## Multi-class condition

In a multi-class condition, a similar approach must be considered, but it must be done per class. A theoretical confusion matrix from our TTR problem:

|  | **Supplementary Figure 2.** Structure of a multi-class confusion matrix according to our TTR problem. |
| --- | --- |

A multi-class problem poses additional challenges because it is difficult to identify the TP, TN, false positive (FP) and false negative (FN) and, consequently, calculate accuracy and sensitivity. However, it is possible to identify each of these structures by focusing on one class at a time (Figure S4). Figure S4 highlights the TP, FP, TN and FN for class A (0% ≤ TTR < 25%), which allows to calculate accuracy and sensitivity for class A:

| ${Accuracy}_{1}=\frac{{TP}_{1}+\sum{TN}_{1}}{P+N}$ | (3) |
| --- | --- |
| ${Sensitivity}_{1}=\frac{{TP}_{1}}{P}$ | (4) |

Similarly, the accuracy and sensitivity must be calculated for the other classes (25-50%, 50-75% and 75-100%). The final accuracy and sensitivity should be calculated as the average of all classes. In our case:

| $Accuracy=\frac{{Accuracy}_{1}+{Accuracy}_{2}+{Accuracy}_{3}+{Accuracy}_{4}}{4}$ | (5) |
| --- | --- |
| $Sensitivity=\frac{{Sensitivity}_{1}+{Sensitivity}_{2}+{Sensitivity}_{3}+{Sensitivity}_{4}}{4}$ | (6) |

**Supplementary Figure 3.** Identification of TP, FP, TN and FN for class A (0% ≤ TTR < 25%) in our multi-class problem.

### Why should sensitivity be preferred over accuracy in this case?

Since the TTR problem is a multi-class problem, the number of samples on TP and TN are imbalanced. Figure S5 illustrates TP (green box) and TN (red box) for class A for the ‘One Week Daytime’ schedule. In particular, TN is always more populated than TP because it aggregates all the data points within all other classes. This will induce a high accuracy in all cases, inflated by the data points in the TN group.

This high accuracy might not represent the true classification performance of the BP modality being tested. Simply put, we are more interested in understanding how many of the actual positives (P) were correctly identified (TP). The latter is, as we saw above, defined by the sensitivity (Figure S6, TP dark green box, P light green box).

| 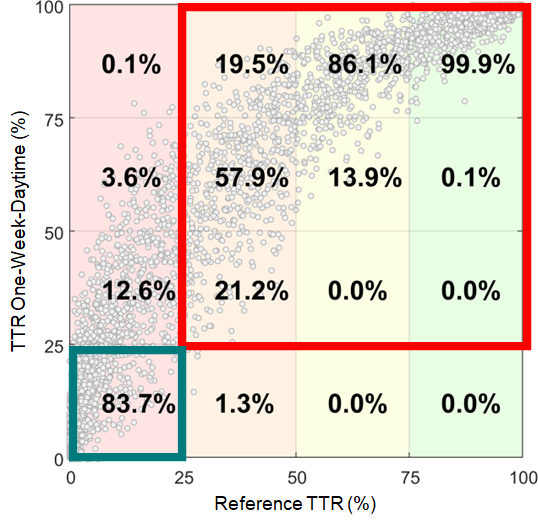 | **Supplementary Figure 4.** Confusion matrix for the ‘One-Week-Daytime’ schedule. The green box represents the TP and the red box represents TN for class A. Notice the data imbalance between these two sub-groups, with TN being more populated. |
| --- | --- |
| 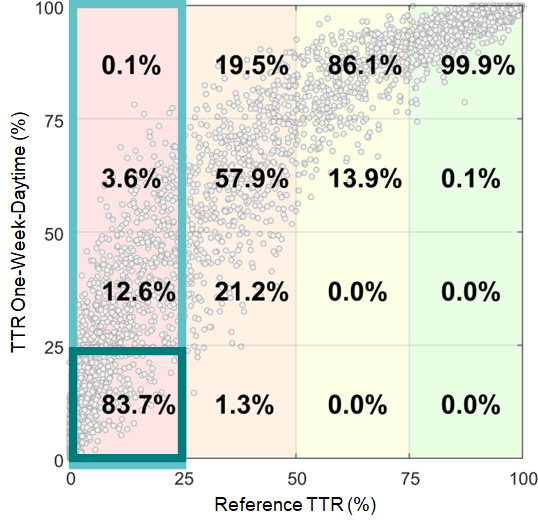 | **Supplementary Figure 5.** Confusion matrix for ‘One-Week-Daytime’ schedule highlighting class A’s TP (dark green box) and P (light green box) for sensitivity calculation. |

# Results

## Number of BP readings per day

A total of 2,252,224 SBP readings were collected during 15 days of consecutive use of the Aktiia monitor for all 5,189 subjects (mean ± SD [IQR]: 29.01 ± 11.4 [23-33] readings per 24-hr per subject, 133.8 ± 15.8 [123-143] mmHg; 19.28 ± 8.8 [14-23] daytime readings per subject; 9.80 ± 4.4 [8-11] night-time readings per subject). One hundred percent of the days had 6 of more measurements and 91.2% of the nights had 6 of more measurements. ‘One Day 24-hr' schedule used a total of 153,367 SBP readings (29.6 ± 12.2 [23-33] readings per 24-hr per subject) for TTR calculation, with average 24-hr SBP 133.9 ± 15.8 [123-143] mmHg. ‘One Week Daytime’ schedule used a total of 707,367 daytime SBP readings for TTR calculation (136.3 ± 49.8 [108-150] daytime readings per consecutive 7-day period per subject, 19.5 ± 8.9 [14-23] daytime readings per 24-hr per subject, with average daytime SBP 135.2 ± 15.8 [125-145] mmHg). Figures S7 to S9 show, respectively, the histogram of BP readings per 24-h during the 15 days used to calculate the reference TTR, the histogram of daytime BP readings during the 15 days, and the histogram of night-time BP readings during the 15 days.


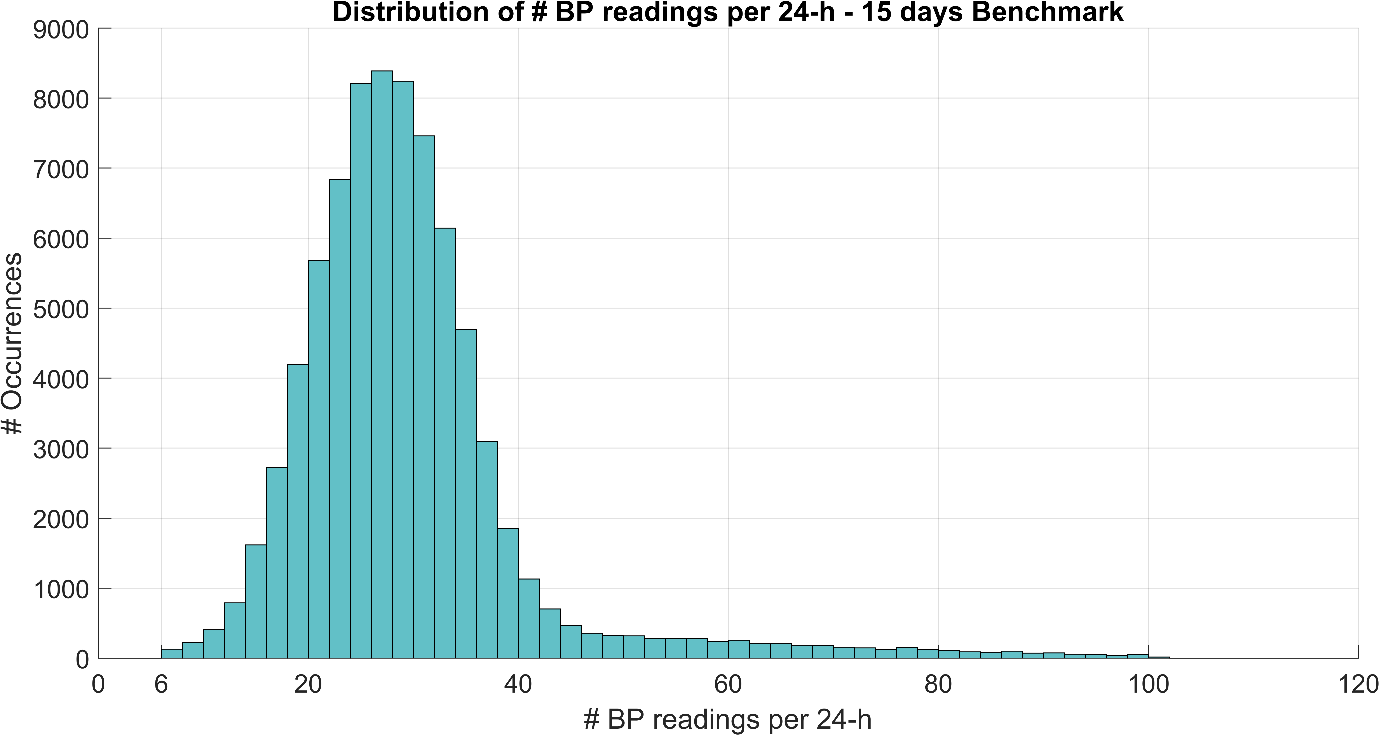


**Supplementary Figure 6.** Distribution of the quantity of BP readings per day performed during the 15 days and used to calculate the reference (Aktiia monitor).


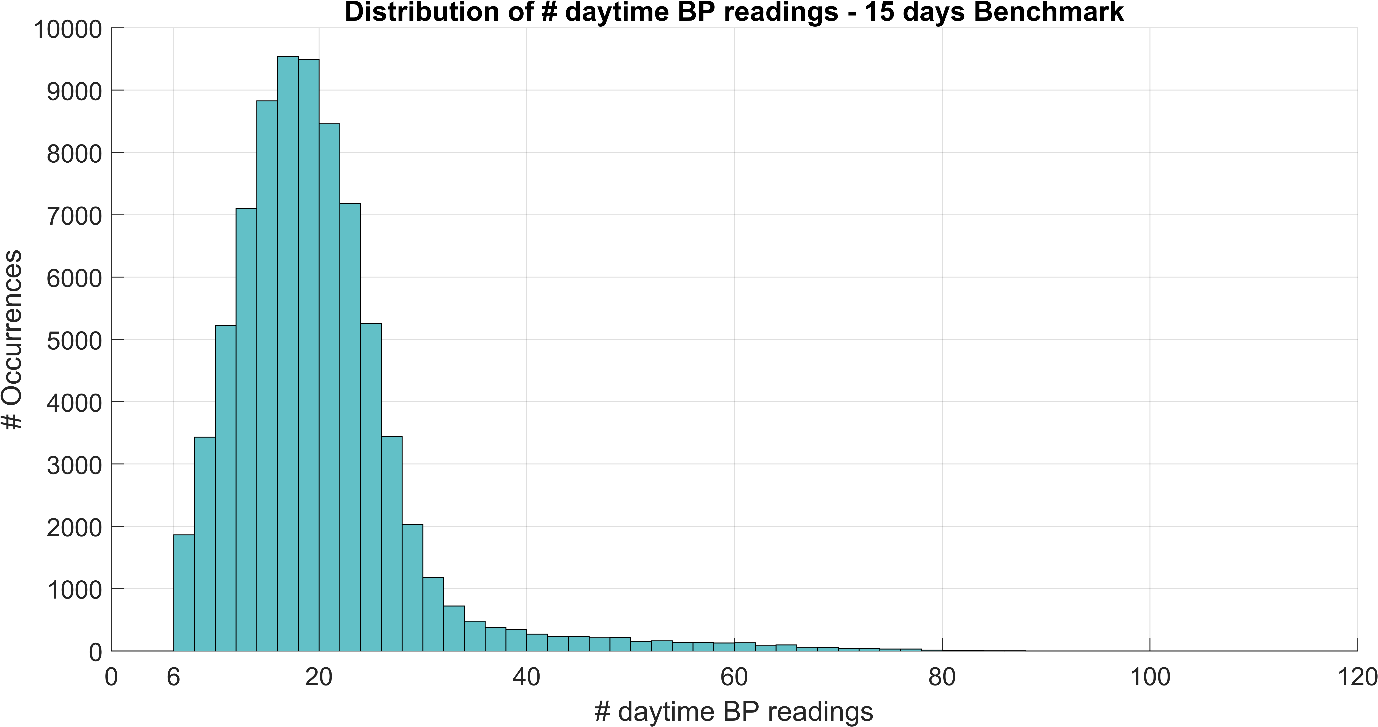


**Supplementary Figure 7.** Distribution of the quantity of daytime BP readings per day performed during the 15 days.


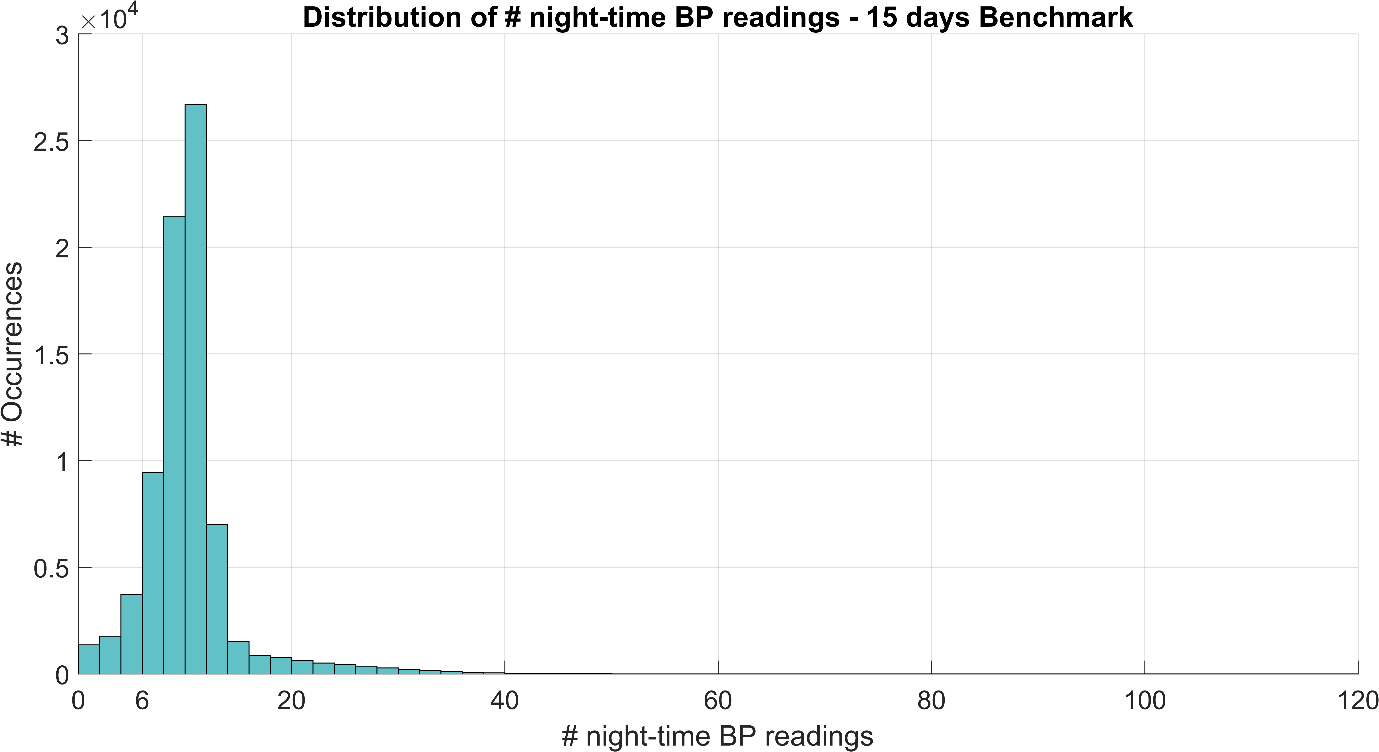


**Supplementary Figure 8.** Distribution of the quantity of night-time BP readings per day performed during the 15 days.

## SBP values

Figure S10 shows the histogram of SBP values used to estimate reference TTR


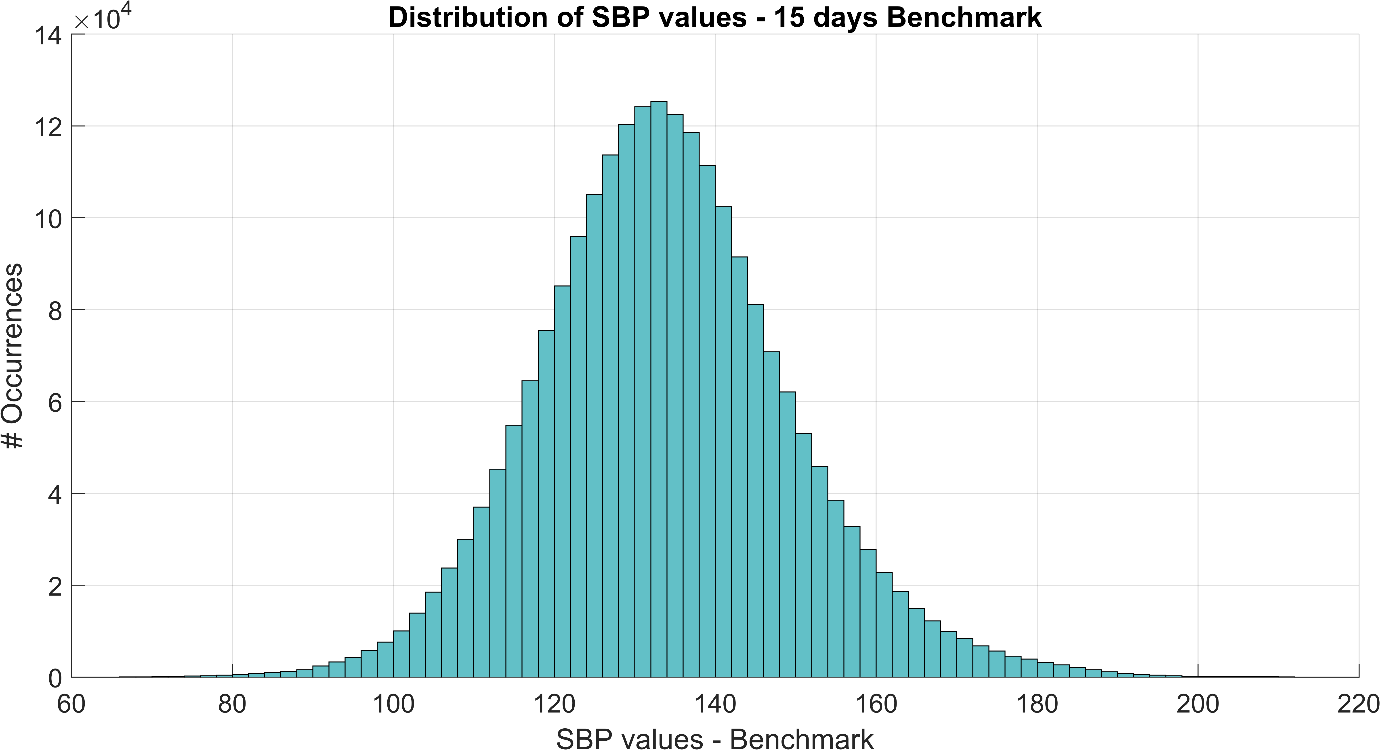


**Supplementary Figure 9.** Distribution of the SBP values used by reference to estimate TTR (Aktiia monitor).

## Additional performance metrics for 15-day BP reference TTR

The accuracy (as opposed to sensitivity), F1-score, kappa score and positive predictive value (PPV) for the TTR calculated under different conditions compared to the 15-day reference TTR are shown in Figure S11. As illustrated previously, accuracy is abnormally high due to class imbalance.

| 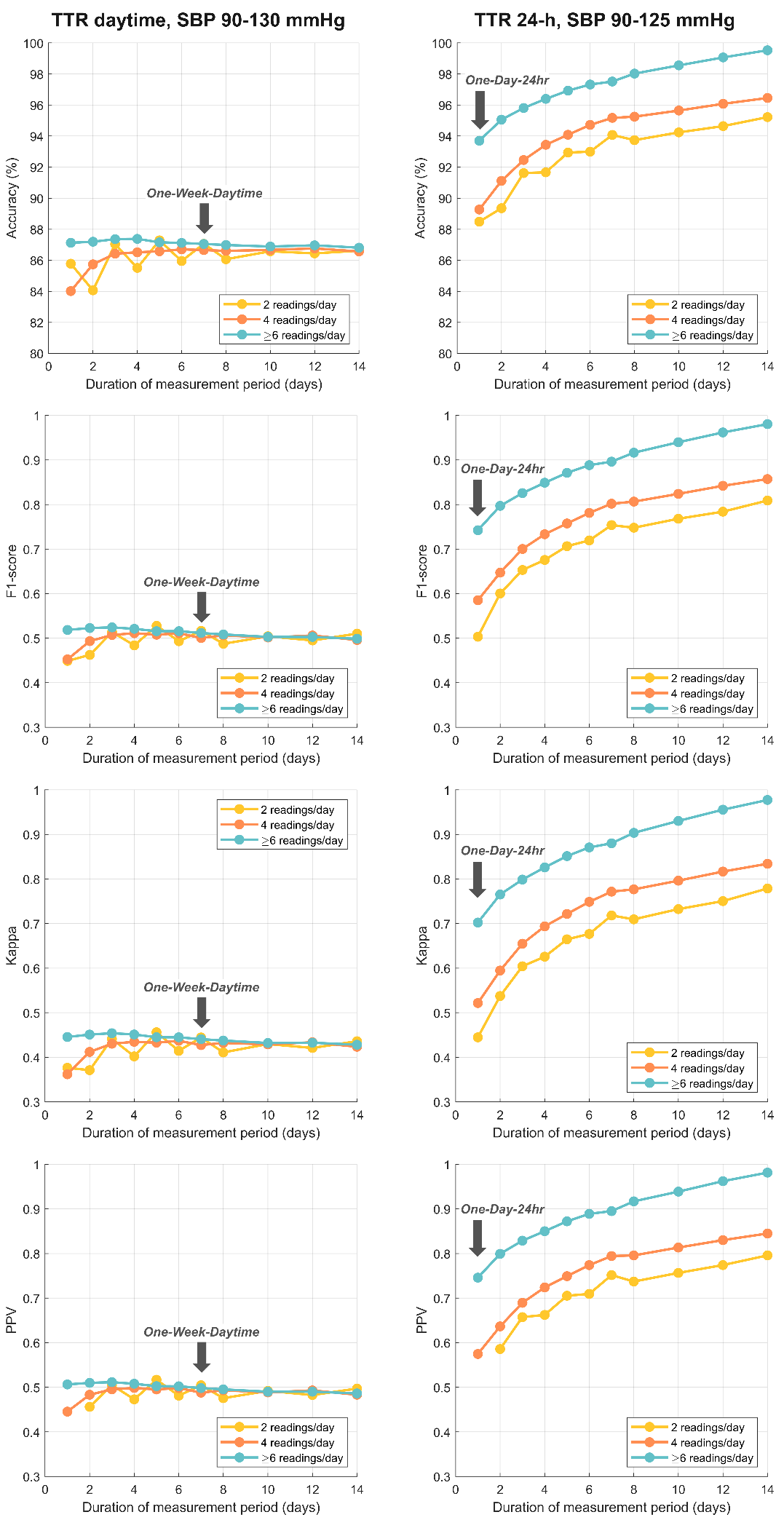 | **Supplementary Figure 10.** Additional classification performance metrics for 15-day BP reference TTR. |
| --- | --- |

## Reference TTR with 30 days

Reference TTR was calculated with 3577 patients that had at least six daily daytime readings in each of 30 consecutive days. Figure S12 illustrates that results were similar to results using 15 consecutive days of data for both daytime (left-hand side) and 24-h (right-hand side) monitoring. Compared to the 30-day reference TTR, sensitivity for ‘One-Week-Daytime’ schedule was 54.5%, and 71.5% for the ‘One-Day-24hr’ schedule. The schedule with seven days of 24-hr continual monitoring reached 87.9% sensitivity compared to the 30-day reference TTR, and 15 days of 24-hr continual monitoring reached 93.0%-93.6% sensitivity compared to the 30-day reference TTR. These results suggest the 15 days-TTR reference should suffice for this retrospective investigation.

**
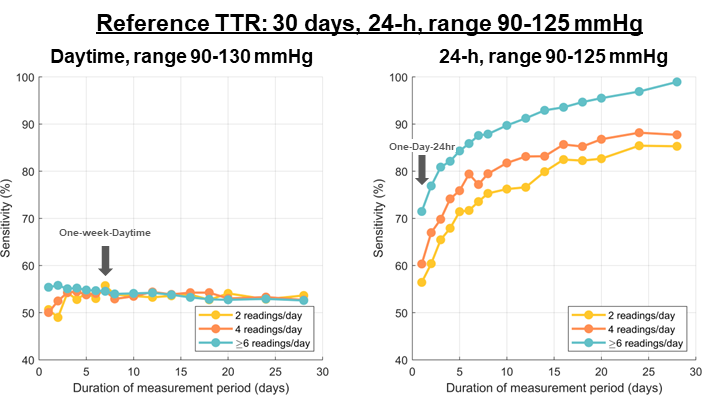
**

**Supplementary Figure 11.** Sensitivity of BP risk group classification, comparing the 30-day reference TTR to different schedules for BP monitoring. Left-hand-side depicts sensitivity for TTR calculated with daytime only SBP for a range of days, and only 2, only 4 and at least 6 readings/day. Right-hand-side depicts sensitivity for TTR calculated with 24-hr SBP data for a range of days, and only 2, only 4 and at least 6 readings/day. Grey arrows highlight the sensitivity for selected layouts: ‘One-Day-24hr’ and ‘One-Week-Daytime’ schedules.
